# Estimulação do Ramo Esquerdo do Sistema His-Purkinje: Experiência Inicial

**DOI:** 10.36660/abc.20201085

**Published:** 2021-11-17

**Authors:** Alexander Romeno Janner Dal Forno, Caique M. P. Ternes, João Vítor Ternes Rech, Helcio Garcia Nascimento, Andrei Lewandowski, Grazyelle Damasceno, Andre d’Avila

**Affiliations:** 1 Hospital SOS Cardio Florianópolis SC Brasil Hospital SOS Cardio , Florianópolis , SC – Brasil

**Keywords:** Marca-Passo Artificial, Estimulação Cardíaca Artificial, Terapia por Estimulação Elétrica

## Abstract

**Fundamento:**

A estimulação ventricular direita convencional aumenta o risco de fibrilação atrial e insuficiência cardíaca em portadores de marca-passo. A estimulação do ramo esquerdo (RE) do sistema His-Purkinje pode evitar os desfechos indesejados da estimulação ventricular direita.

**Objetivo:**

Analisar retrospectivamente os desfechos intraoperatórios, eletrocardiográficos e os dados clínicos do seguimento inicial de pacientes submetidos à estimulação do RE.

**Métodos:**

Foram avaliados os parâmetros eletrônicos do implante e eventuais complicações precoces de 52 pacientes consecutivos submetidos à estimulação do sistema de condução. O nível de significância alfa adotado foi igual a 0,05.

**Resultados:**

52 pacientes foram submetidos a estimulação do RE do sistema His-Purkinje, obtendo sucesso em 50 procedimentos. 69,2% dos pacientes eram do sexo masculino e a mediana e intervalo interquatil da idade no momento do implante foi de 73,5 (65,0-80,0) anos. A duração do QRS pré-implante foi de 146 (104-175) ms e de 120 (112-130) ms após o procedimento. O tempo de ativação do ventrículo esquerdo foi de 78 (70-84) ms. A amplitude da onda R foi de 12,00 (7,95-15,30) mV, com limiar de estimulação de 0,5 (0,4-0,7) V × 0,4 ms e impedância de 676 (534-780) ohms. O tempo de procedimento foi de 116 (90-130) min e o tempo de fluoroscopia foi de 14,2 (10,0-21,6) min.

**Conclusão:**

A estimulação cardíaca do sistema de condução His-Purkinje por meio da estimulação do ramo esquerdo é uma técnica segura e factível. Nesta casuística, apresentou alta taxa de sucesso, foi realizada com tempo de procedimento e fluoroscopia baixos e obteve medidas eletrônicas adequadas.

## Introdução

A estimulação ventricular direita é a modalidade de estimulação mais utilizada em todo o mundo para correção de distúrbios da condução atrioventricular (AV). Entretanto, este tipo de estimulação aumenta o risco de fibrilação atrial, pode piorar a classe funcional de insuficiência cardíaca (CFIC) e aumentar a necessidade de hospitalização por insuficiência cardíaca (IC) em até 20% dos pacientes em 4 anos.^1 - 3^

O risco desses eventos adversos aumenta quando a estimulação ventricular se faz necessária > 40% do tempo e em pacientes com disfunção ventricular prévia ao implante, principalmente quando a duração do QRS estimulado excede 150 ms. Vários sítios alternativos já foram explorados na tentativa de evitar os efeitos deletérios da estimulação ventricular muscular, sem uma comprovação real de benefício clínico ou ecocardiográfico.^4^ Nesses pacientes, a terapia de ressincronização cardíaca (TRC) pode amenizar ou reverter os efeitos deletérios da estimulação ventricular direita ao reduzir a dissincronia intra e interventricular, tal como ocorre em pacientes com bloqueio de ramo esquerdo (BRE).^5^

Como alternativa à TRC convencional, pode ser utilizada a estimulação do sistema de condução His-Purkinje em qualquer uma de suas porções, tanto inicial como no feixe de His ou mais distal, como no ramo esquerdo (RE). Na estimulação do feixe de His, o eletrodo ventricular direito é fixado próximo ao ápice do Triângulo de Koch, permitindo a captura seletiva – ou não – do sistema His-Purkinje proximal. Dessa forma, ao recrutar o sistema de condução do coração, restabelece a fisiologia normal da ativação ventricular, prevenindo os efeitos indesejados da estimulação convencional.^3 , 6^

Essa técnica, no entanto, apresenta limitações. Em muitos casos, a energia necessária para capturar o feixe de His é muito alta quando comparada com a energia necessária à estimulação convencional do ventrículo direito, resultando em uma depleção rápida da bateria do marca-passo (MP) principalmente em pacientes com bloqueios intra ou infra-hissiano e bloqueio distal de RE. Além disso, a atividade ventricular intrínseca medida pelo dispositivo (onda R) pode ser de muito baixa amplitude na posição do feixe de His, dificultando a programação do MP.^7^

Como uma alternativa à técnica de estimulação do feixe de His, em 2017, Huang et al. descreveram o primeiro caso de estimulação do sistema de condução diretamente no RE em paciente inelegível para estimulação fisiológica do feixe de His.^8^ Nessa abordagem, o eletrodo ventricular direito (geralmente o mesmo eletrodo que se destina ao implante no feixe de His) é fixado profundamente no septo interventricular, atingindo a região subendocárdica do ventrículo esquerdo, permitindo a estimulação direta do sistema de condução His-Purkinje através da ativação do RE. Essa modalidade de estimulação resulta em complexos QRS com duração equivalente à da estimulação do feixe de His, com menor limiar de captura e onda R adequada, facilitando a programação do MP e permitindo uma durabilidade da bateria semelhante à dos MP convencionais.^7^

O presente estudo tem por objetivo apresentar o resultado cirúrgico e eletrocardiográfico imediato e do seguimento clínico precoce dos primeiros 52 pacientes submetidos a implante de MP para estimulação do sistema de condução com estimulação direta do ramo esquerdo do sistema His-Purkinje em um centro de referência em eletrofisiologia.

## Métodos

**Figura 1:**
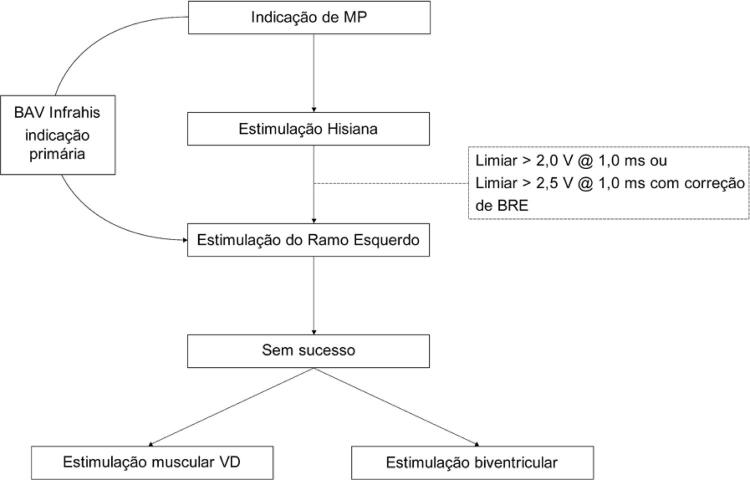
– Fluxograma de estimulação do sistema His-Purkinje distal – ramo esquerdo. Pacientes com indicação de marca-passo que apresentavam QRS estreito ou bloqueio em nível nodal e apresentavam limiares de captura do His maior que 2 V X 1,0 ms ou maior que 2,5 V X 1,0 ms para correção de bloqueios de ramo preexistente eram encaminhados para implante mais distais junto ao ramo esquerdo (RE) (implante secundário). Pacientes com bloqueio intra ou infra-hissianos documentados por estudo eletrofisiológico, eram encaminhados diretamente para estimulação distal (chamado implante primário). Nos casos de insucesso, o paciente era encaminhado para estimulação convencional septal ou estimulação biventricular. MP: marca-passo; BAV: bloqueio atrioventricular; BRE: bloqueio do ramo esquerdo; VD: ventrículo direito.

Trata-se de um estudo descritivo, retrospectivo em um centro para avaliação de viabilidade de um sítio alternativo de estimulação do sistema de condução His-Purkinje. Foram consideradas quatro categorias de inclusão para o estudo: implante primário, implante secundário, limiar elevado de MP hissiano (>2,0 V x 1,0 ms) e ressincronização ( Figura 1 ). Foram considerados implantes primários todos os pacientes em que a intenção inicial da intervenção era a estimulação do RE do sistema His-Purkinje, e secundários todas as intervenções em que a intenção inicial era a estimulação do feixe de His e não houve sucesso devido a limiar elevado transoperatório (limiar >2,0 V ou pacientes com BRE com limiar >2,5 V com correção do bloqueio).

Foram incluídos 52 pacientes consecutivos submetidos a implante de MP com estimulação do RE em um centro de referência. Todos os pacientes assinaram o termo de consentimento livre e esclarecido (TCLE) antes do procedimento. Este é um estudo retrospectivo com coleta de informações dos registros médicos do implante no Hospital SOS Cárdio realizado de acordo com a Declaração de Helsinki e aprovado pelo Comitê de Ética do Instituto de Cardiologia do estado de Santa Catarina por meio do Certificado de Apresentação de Apreciação Ética nº 36517420.7.0000.0113 com parecer favorável nº 4.293.667. Foram avaliados sucesso agudo do procedimento, complicações precoces e necessidade de reintervenção. Os pacientes incluídos no estudo tinham indicação de implante de MP de acordo com a Diretriz Brasileira de Dispositivos Cardíacos Eletrônicos Implantáveis (DCEI) e do ACC/AHA/HRS de 2018, submetidos ao procedimento de estimulação de RE entre agosto de 2019 e novembro de 2020 em um centro médico de referência.

### Técnica de implante do eletrodo septal para estimulação do ramo esquerdo

**Via de acesso:** Todos os procedimentos foram realizados sob anestesia geral. A punção da veia axilar guiada por fluoroscopia com ou sem a injeção de contraste na veia cubital esquerda foi a via de acesso preferida. Em um dos pacientes, portador de MP há 8 anos, foi observada oclusão total da veia subclávia esquerda. Nesse caso, o implante foi realizado através da veia axilar direita e o eletrodo tunelizado até a loja subpeitoral esquerda. Nos demais pacientes, o acesso axilar esquerdo foi obtido sem complicações.

**Figura 2:**
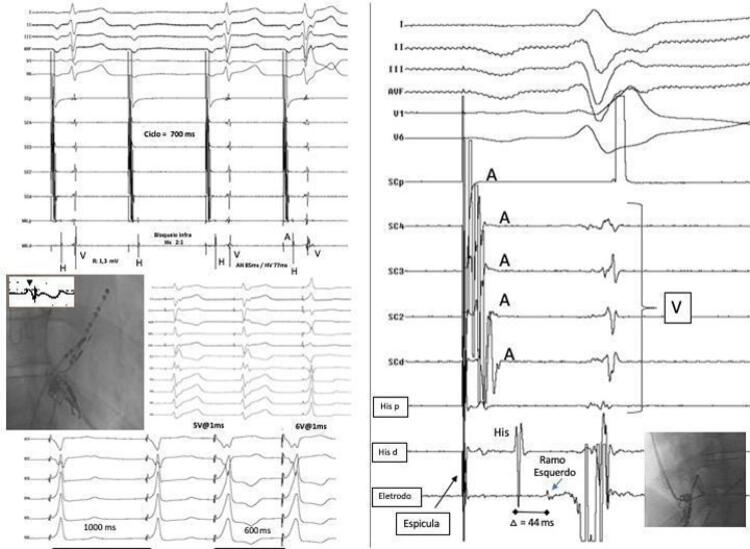
– Técnica de implante do eletrodo para captura do ramo esquerdo após tentativa de captura do eletrograma de His. O painel superior esquerdo mostra bloqueio atrioventricular (BAV) infra-hissiano 2:1 durante estimulação atrial contínua com ciclo de 700 ms. Nos batimentos conduzidos, observa-se intervalo AH = 85 ms e HV de 77 ms. No painel central, à esquerda, observa-se cateter decapolar no seio coronariano (SC) que foi utilizado para estimulação atrial e cateter quadripolar na posição de His na posição oblíqua anterior direita a 30°. Esse caso foi realizado concomitantemente com a realização de um estudo eletrofisiológico, e podemos visualizar um cateter decapolar no seio coronário. O cateter quadripolar foi utilizado como referência para posicionamento do eletrodo de marca-passo (MP) na posição do feixe de His – pode ser observada corrente de lesão no eletrograma intracavitário. Durante a estimulação contínua do eletrodo do His, observa-se captura hissiana seletiva sem correção do bloqueio de ramo direito com energia de 5 V e largura de pulso de 1 ms. Com o aumento da energia de estimulação de 5 para 6 V, ocorre captura não seletiva do His com correção do bloqueio de ramo direito com ciclo de estimulação de 1.000 ms. No entanto, o bloqueio volta a ocorrer quando o ciclo de estimulação foi diminuído para 600 ms. Por essa razão (limiar alto e persistência do bloqueio de ramo com frequência cardíaca de 100 bpm [600 ms]), optou-se pelo implante do mesmo eletrodo na região septal mais baixa (painel inferior direito mostrando projeção em oblíquo anterior esquerda a 30 graus). Observa-se registro do eletrograma de His no cateter quadripolar e eletrograma do ramo esquerdo (RE) no eletrodo do marca-passo [E1]) 44 ms depois, sugerindo que o bloqueio estivesse entre o His e o local em que foi registrado o potencial do RE. A: eletrograma atrial; p: proximal; d: distal.

**Bainha e eletrodo para estimulação do ramo esquerdo:** Após a punção venosa, uma bainha Medtronic C315HIS^®^ 69 cm (Medtronic, Minneapolis – MN) de curva fixa era avançada até o átrio direito com auxílio de um fio-guia longo (180 cm). Um eletrodo Medtronic *Select Secure* ^®^ modelo 3830 era posicionado, através da bainha, no nível do anel atrioventricular direito, após discreta rotação anti-horária. O polo distal de eletrodo 3830 *Select Secure* ^®^ Medtronic era exposto para permitir o mapeamento unipolar do eletrograma do feixe de His ( Figura 2 ). Excepcionalmente, foram utilizados cateteres de eletrofisiologia concomitantemente nos casos que necessitavam a realização de estudo eletrofisiológico para confirmar o nível do bloqueio (2 casos). Essa posição era fixada em fluoroscopia na posição oblíqua anterior direita 30° (OAD) e mantida como referência durante o mapeamento da região septal direita. A partir dessa posição e após leve rotação horária, a bainha e o eletrodo 3830 eram, então, avançados através da válvula tricúspide cerca de 1 a 1,5 cm inferoapicalmente em relação à da posição em que o eletrograma do feixe de His havia sido registrado.

**Figura 3:**
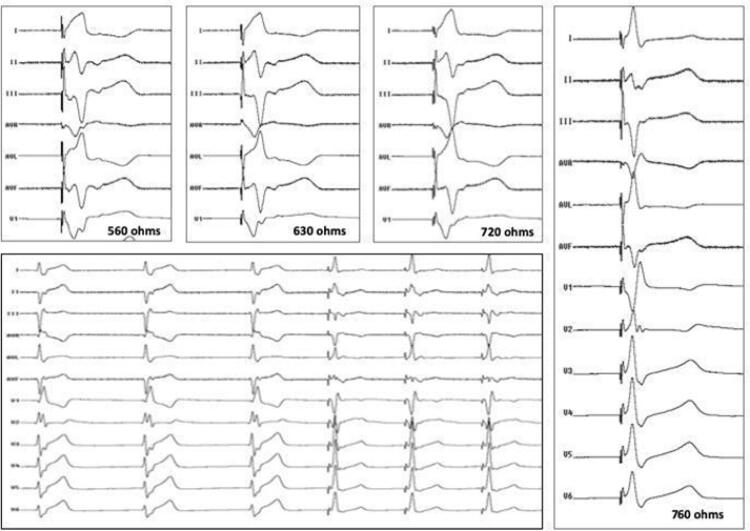
– Variação da impedância durante posicionamento do eletrodo de estimulação para captura do ramo esquerdo. Nota-se aumento progressivo da impedância de 560 para 760ohms acompanhado de aumento progressivo da onda R em V1. A medida inicial mostra complexo QRS em V1 com morfologia em “w”. À medida que o eletrodo avança no septo interventricular em direção à região subendocárdica do septo interventricular esquerdo, a onda R’ em V1 e a impedância durante estimulação unipolar aumentam progressivamente. O eletrocardiograma de 12 derivações ilustra a morfologia final do complexo QRS.

Nos 10 primeiros casos, foi realizada estimulação unipolar em áreas supostamente favoráveis à entrada do eletrodo septal definida pela presença de complexo QRS com morfologia de “W” na derivação V1 (e/ou estreitamento do QRS com padrão de onda R predominante na derivação DI, Rs na derivação DII e rS derivação DIII [ Figura 3 ]).^8^ Entretanto, na experiência dos autores, tal morfologia do complexo QRS podia ser obtida em várias posições do septo interventricular em um mesmo paciente, enquanto, em outros, tal padrão do QRS não poderia ser observado, mesmo após extenso mapeamento do septo interventricular direito. Por esta razão, nos 42 casos subsequentes, o local selecionado para entrada do eletrodo foi definido apenas pela posição do eletrodo no septo interventricular em relação à referência do local onde um eletrograma de His havia sido registrado.

Nesses locais, mais rotação anti-horária era aplicada à bainha até que um ângulo próximo a 90° entre o septo interventricular e a bainha C315HIS^®^ fosse observado na projeção oblíqua anterior esquerda (OAE) 30°. Uma vez atingida essa posição, o eletrodo 3830 era gentilmente rodado em sentido horário até sua fixação e leve penetração do eletrodo distal (mecanismo de fixação ativa) no septo interventricular. À medida que inserções progressivas do eletrodo eram visualizadas, era realizada estimulação unipolar para avaliar a duração e morfologia do complexo QRS estimulado, o eletrograma local (quando um QRS intrínseco era disponível) e a medida da impedância. À medida que o eletrodo penetra o septo interventricular, a impedância aumenta gradativamente durante a estimulação unipolar ( Figura 3 ).

O eletrodo era, então, avançado através de rotação horária até atingir profundidade de cerca de 1 cm em relação à extremidade distal da bainha, justaposta ao septo interventricular, na posição oblíqua anterior esquerda a 30° (OAE). A partir desse ponto, o eletrodo 3830 era conectado ao polígrafo de eletrofisiologia (EP Tracer^®^ Schwarzer Cardiotek, NL) e ao analisador do MP para registro do eletrograma local e a medida da onda R. A morfologia, a duração do complexo QRS, e a variação da impedância – que aumenta gradativamente à medida que o eletrodo penetra o septo interventricular – eram comparados àquelas obtidas no ponto de entrada do eletrodo durante estimulação com energia de 3,0 V × 0,4 ms. Se não houvesse critério de captura do RE, o eletrodo era lentamente avançado com rotação horária adicional de meia a uma volta, e as medidas repetidas até que esses critérios fossem obtidos.

**Critérios para definir a captura do ramo esquerdo:** Uma das formas mais aceitas atualmente como critério de captura do ramo esquerdo é a medida do tempo de despolarização completa do ventrículo esquerdo, chamado de tempo de ativação do ventrículo esquerdo (TAVE). Eletrocardiograficamente, o TAVE corresponde ao intervalo em milissegundos entre a espícula de estimulação e o pico da onda R em V4, V5 ou V6; quando menor que 90 ms, caracteriza uma despolarização do VE rápida e através do sistema de condução. Nos pontos em que era obtido complexo QRS com morfologia sugestiva de captura do RE (morfologia qR ou rsR’ em V1), era realizada estimulação com baixa e alta energia (respectivamente, 1,5 V × 0,4 ms e 10 V × 0,4 ms) com o intuito de observar redução adicional da duração do complexo QRS e TAVE em milissegundos. Se o local fosse considerado inadequado, o eletrodo seria avançado por mais alguns milímetros até que não houvesse diminuição desses intervalos durante estimulação com alta energia.

**Figura 4:**
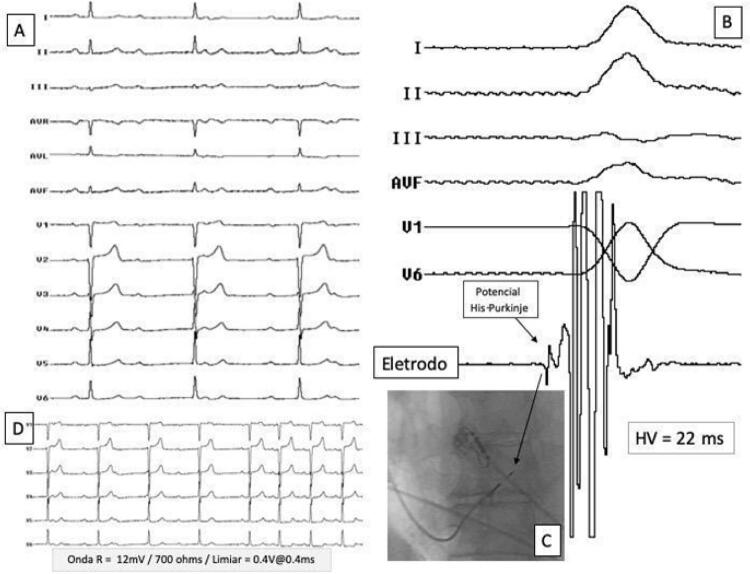
– Implante de marca-passo para captura do ramo esquerdo em paciente portadora de bloqueio atrioventricular total (BAVT). Paciente do sexo feminino com BAVT e escape com complexo QRS estreito (painel A), submetida a implante de marca-passo (MP) para captura do ramo esquerdo (RE). No painel B, observam-se derivações eletrocardiográficas e o registro intracavitário do potencial do RE o com HV=22 ms. A posição do eletrodo do MP posicionado profundamente no septo interventricular pode ser observada no painel C em oblíqua anterior esquerda a 30 graus. Neste local, a onda R era de 12mV com limiar de estimulação de 0,4 VX0,4 ms, resultando em um QRS estimulado estreito, idêntico ao do ritmo de escape, conforme evidenciado pelas derivações precordiais (V1 a V6) no painel D.

**Manobras para avaliar a captura do ramo esquerdo:** Definiu-se como captura do RE a presença de um padrão eletrocardiográfico característico em V1 (qR ou rqR’) e pelo menos um dos seguintes critérios: 1) Identificação de potencial do RE/Purkinje precedendo o complexo QRS, com intervalo do potencial local até eletrocardiograma de superfície entre 10 e 50 ms ( Figura 4 ); 2) manutenção do TAVE <90 ms durante estimulação unipolar com alta e baixa energia; 3) mudança progressiva entre o padrão não seletivo e seletivo de captura do RE durante estimulação unipolar contínua com diferentes níveis de energia; 4) mudança do padrão não seletivo para um padrão muscular septal quando muito próximo ao limiar final de captura; e, por último, 5) teste com extraestímulos com resposta muscular pura ou de captura seletiva do RE.^9^

**Figura 5:**
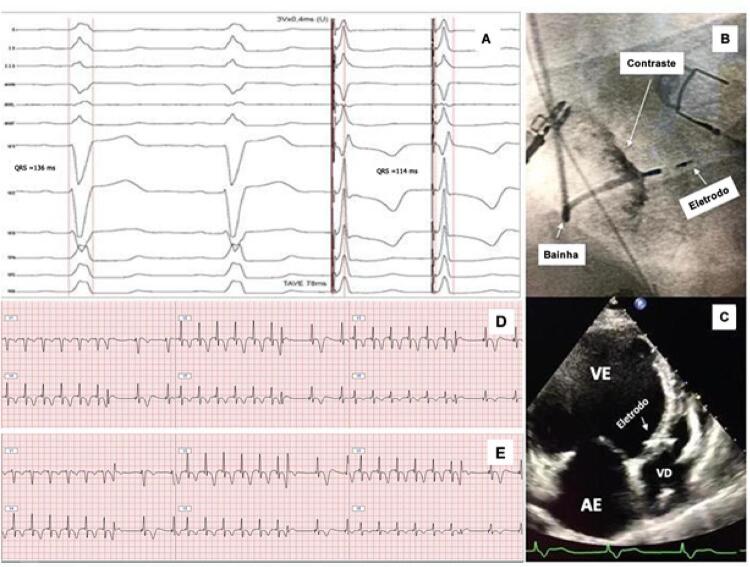
– Técnica de extraestímulos por meio do eletrodo do marca-passo para demonstrar captura do ramo esquerdo (RE). O painel A mostra a presença de ritmo sinusal com bloqueio do ramo esquerdo (BRE) com duração do complexo QRS de 136 ms (dois batimentos iniciais). Durante a estimulação do RE, observa-se redução da duração do complexo QRS para 114 ms com um tempo de ativação do ventricular esquerdo (TAVE) de 78 ms. A posição do eletrodo pode ser evidenciada em projeção oblíqua anterior esquerda a 30° durante injeção de contraste através da bainha, e um ecocardiograma transtorácico bidimensional ilustra a posição desse mesmo eletrodo por meio do septo interventricular no painel C. Os painéis D e E ilustram a diferença na morfologia do complexo QRS em V1 durante estimulação contínua seguida de extraestímulos com intervalo de acoplamento progressivamente mais curto. Ao contrário do que demonstrado no painel E, em que um complexo qR pode ser claramente identificado, o complexo QRS em V1 não apresenta a morfologia característica de captura do RE. No painel D, o período refratário da porção muscular do septo interventricular ainda não havia sido atingido. Com a diminuição do acoplamento do extraestímulo em 10 ms, observa-se a perda da captura muscular septal e o padrão típico de captura do RE. VD: ventrículo direito; VE: ventrículo esquerdo; AE: átrio esquerdo.

**Teste com extraestímulos:** A proximidade entre o valor do limiar de estimulação do RE e do músculo cardíaco que o circunda ocasionalmente pode dificultar a distinção entre a captura do RE e a captura ventricular septal esquerda, pois o complexo QRS estimulado pode representar uma fusão dos padrões de estimulação dessas duas estruturas. Por esta razão, utilizamos rotineiramente o teste de extraestímulos descrito por Jastrzbski.^10^ O teste consiste em aplicar, com auxílio do polígrafo de eletrofisiologia, um ciclo fixo de estimulação ventricular unipolar de 8 batimentos consecutivos no eletrodo destinado ao RE, seguido de um extraestímulo com acoplamento progressivamente mais curto. A segunda forma do teste consiste na deflagração de um extraestímulo isolado, também, com acoplamento progressivamente mais curto. Conclui-se que há captura do RE quando ocorre mudança na morfologia do complexo QRS, de morfologia de captura não seletiva do RE para captura muscular pura ou captura seletiva do sistema de condução.^9^ Ambas as respostas são diagnósticas, conforme ilustrado na Figura 5 . Outra forma de confirmação de captura do RE é a realização do teste de limiar com larguras de pulso muito pequenas, como 0,1 ou 0,05 ms.

**Retirada da bainha e conexão do eletrodo septal ao gerador:** Uma vez confirmada a captura do RE, é realizada injeção de pequena quantidade de contraste através da bainha C315, para contrastar a borda do septo interventricular direito e documentar a profundidade atingida pelo eletrodo no trajeto intraseptal. Nesse momento, a bainha era recuada até o átrio direito, as medidas eletrônicas repetidas e a bainha finalmente retirada conforme orientações do fabricante.

**Conectando os eletrodos ao gerador:** Nos casos de BAVT com escape com QRS alargado, devido ao risco de apresentar assistolia durante a manipulação intraseptal, o eletrodo inicialmente destinado ao átrio era fixado ao ventrículo direito e utilizado para estimulação provisória ventricular e após o implante do eletrodo intraseptal, levado ao apêndice auricular direito. Nos casos de MP bicameral e ressincronizador, o eletrodo 3830 era conectado à porta ventricular direita do dispositivo. No único caso em que o MP do ramo do esquerdo foi utilizado em um desfibrilador, o eletrodo 3830 foi conectado na porta do ventrículo direito através da conexão IS1/DF1.

Nos casos de MP dupla câmara ou de TRC, o eletrodo atrial e o do ventrículo esquerdo (seio coronariano) foram posicionados com a técnica habitual.

**Análise estatística:** As variáveis incluídas na análise foram sexo, idade, comorbidades, cardiopatia de base, tipo de bradiarritmia, tempo médio de *follow-up* , duração do complexo QRS pré e pós-implante, TAVE, tipo de indicação do procedimento, tempo de procedimento e fluoroscopia, bem como o limiar de captura (valor do limiar unipolar da estimulação × largura de pulso em Volts), impedância do eletrodo e amplitude da onda R. Os parâmetros elétricos dos dois pacientes nos quais a captura do RE não foi possível foram excluídos dessa análise.

A normalidade dos dados foi avaliada por meio do teste de Shapiro-Wilk. As variáveis contínuas de parâmetros do implante apresentaram distribuição não normal e foram comparadas com teste não paramétrico de Wilcoxon. Os resultados foram descritos em mediana e intervalo-interquartil (IQ) Q1 – Q3 (percentil 25 – percentil 75). Foi considerado como estatisticamente significativo um valor de p bicaudal <0,05. Todas as análises foram realizadas utilizando o programa R versão 3.6.2.

## Resultados

### Características dos pacientes

Foram realizados 52 procedimentos. A mediana e IQ da idade dos pacientes foi de 73,5 (65,0-80,0) anos e 69,2% eram do sexo masculino. Quarenta pacientes apresentavam cardiopatia não isquêmica (76,92%), 4 apresentavam cardiopatia isquêmica (7,69%) e 8 apresentavam cardiopatia induzida por MP prévio (15,38%). A característica do tipo de bradiarritmia dos pacientes está descrita na Tabela 1 , e um mesmo paciente pode apresentar mais de um tipo de bradiarritmia. Sete pacientes com FA necessitaram de MP para controle da frequência após BAVT. Em 63,5% dos implantes (n=33), a intenção inicial do procedimento foi o implante primário de MP para estimulação do RE. Em 13,5% dos pacientes (n=7), houve a tentativa inicial de estimulação do feixe de His, e devido a medidas insatisfatórias obtidas no transoperatório (limiar de estimulação >2 V x 1 ms e/ou onda R <2 mV), optou-se pela estimulação do RE – considerados implantes secundários. Em 7,7% dos pacientes (n=4) com MP hissiano prévio, houve aumento do limiar de comando > 4,0 V x 1 ms durante o seguimento clínico, e 15,4% dos pacientes (n=8) não responderam à TRC convencional, tendo sido submetidos à estimulação de RE. Foi obtido sucesso em 50/52 pacientes (96,2%), taxa semelhante à demonstrada na literatura.^11^ Os dados dos dois pacientes nos quais não foi obtida captura do RE não foram incluídos nas análises seguintes. Dezessete pacientes eram portadores de cardiopatia estrutural com fração de ejeção do ventrículo esquerdo (FEVE) menor que 50%. Dos dispositivos implantados, 8 foram ressincronizadores (15,4%), 3 MP unicamerais (5,8%), 3 cardiodesfibriladores bicamerais (5,8%) e 38 MP bicamerais (73,1% dos dispositivos).

**Tabela 1:** – Características demográficas dos pacientes submetidos à estimulação de ramo esquerdo

	Total (N=52)
**Sexo,** *n (%)*	
Masculino	36 (69,2%)
Feminino	16 (30,8%)
**Idade (anos),** *mediana (IQ)*	73,5 (65,0-80,0)
**HAS,** *n (%)*	39 (75,0%)
**DM,** *n (%)*	17 (32,7%)
**CFIC,** *n (%)*	
NYHA I-II	11 (21,2%)
NYHA III-IV	41 (78,8%)
**Cardiopatia de base,** *n (%)*	
Não isquêmica	40 (76,92%)
Isquêmica	4 (7,69%)
Induzida por MP prévio	8 (15,38%)
**Tipo de bradiarritmia,** *n (%)* ^¶^	
Doença do nó sinusal	3 (5,8%)
BAV 1º grau	4 (7,7%)
BAV 2º grau	7 (13,5%)
BAV 3º grau	24 (46,2%)
FA + BAVT	5 (9,6%)
**FEVE Basal,** *mediana (IQ)*	56,2 (44,8-67,1)
**Indicação,** *n (%)*	
Implante primário	33 (63,5%)
Implante secundário	7 (13,5%)
Limiar elevado de MP hissiano	4 (7,7%)
TRC	8 (15,4%)
**Desfecho,** *n (%)*	
Sucesso	50 (96,2%)
Falha	2 (3,8%)

*¶: Os pacientes podem apresentar mais de um tipo de bradiarritmia. HAS: hipertensão arterial sistêmica; DM: diabetes melito; BAV: bloqueio atrioventricular; FA: fibrilação atrial; BAVT: bloqueio atrioventricular total; MP: marca-passo; TRC: terapia de ressincronização cardíaca; CFIC: classe funcional de insuficiência cardíaca; NYHA: New York Heart Association; FEVE: fração de ejeção do ventrículo esquerdo.*

**Figura 6:**
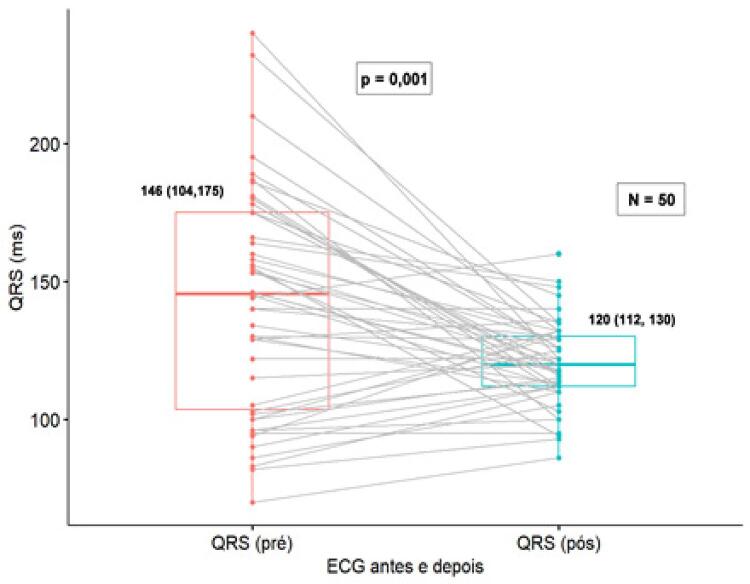
– Duração do complexo QRS antes e após estimulação direta do ramo esquerdo. Utilizado teste não paramétrico de Wilcoxon (p=0,001).

**Medidas eletrônicas:** A mediana e IQ da duração do QRS pré-operatório foi de 146 (104-175) ms. A duração do complexo QRS durante a estimulação do RE foi de 120 (112-130) ms ( Figura 6 ) com um TAVE de 78 (70-84) ms. O tempo médio de procedimento foi de 116 (90-130) minutos com um tempo médio de fluoroscopia de 14,2 (10,0-21,6) min. O limiar da estimulação para captura do RE foi de 0,5 (0,4-0,7) V × 0,4 ms com uma impedância durante a estimulação de 676 (534-780) ohms e uma onda R de 12,00 (7,95-15,30) mV. Em cinco pacientes com BAVT sem escape, não foi possível avaliar a amplitude da onda R.

**Implantes sem sucesso:** Em dois (2/52) pacientes (3,8%) portadores de miocardiopatia dilatada, não foi possível obter critérios de captura do RE. No primeiro caso, o paciente apresentava diâmetros cavitários aumentados e FEVE de 38%. Houve dificuldade em manipular a bainha C315, impossibilitando o mapeamento do feixe de His. Após diversas tentativas, fratura de duas bainhas e tempo de procedimento e fluoroscopia prolongados, optamos pelo implante de um ressincronizador convencional com eletrodo ventricular esquerdo no ramo posterolateral do seio coronário.

No segundo paciente, com diâmetro diastólico do VE de 59 mm e FEVE de 35%, sem implante prévio de MP e com complexo QRS alargado (BAVT infra-hissiano), não foi possível demostrar nenhum dos critérios de captura do RE apesar do difícil posicionamento e aparentemente posição adequada do eletrodo ventricular. O eletrodo foi substituído por um eletrodo convencional de fixação ativa, posicionado na região médio-septal do ventrículo direito, por receio de deslocamento devido ao grande número de rotações do eletrodo na tentativa de alcançar o RE.

**Tabela 2:** – Resultados dos procedimentos e parâmetros eletrônicos dos marca-passos

	Total (N=50)
QRS pré-operatório (ms), mediana (IQ)	146 (104-175)
QRS pós-operatório (ms), mediana (IQ)	120 (112-130)
TAVE (ms), mediana (IQ)	78 (70-84)
Tempo de fluoroscopia (min), mediana (IQ)	14,2 (10,0-21,6)
Tempo de procedimento (min), mediana (IQ)	116 (90-130)
Limiar (V x 0,4 ms), mediana (IQ)	0,5 (0,4-0,7)
Impedância (ohms), mediana (IQ)	676 (534-780)
Onda R (mV), mediana (IQ)	12,00 (7,95-15,30)

*TAVE: tempo de ativação do ventrículo esquerdo.*

**Seguimento clínico**: Durante o seguimento [mediana e (IQ)] de 8 (3,25-10,00) meses, foi observado deslocamento do eletrodo septal para a porção subtricúspide do ventrículo direito em um paciente que apresentava, ao final do seu procedimento, parâmetros completamente normais. Esse paciente permaneceu assintomático por 30 dias quando, durante o retorno ambulatorial, foi observado complexo QRS alargado e a radiografia de tórax confirmou o deslocamento. O paciente foi submetido novamente ao implante de estimulação do RE e manteve-se clinicamente estável, com parâmetros estáveis de captura do RE até sua última consulta de seguimento 6 meses após o reimplante. Nesse caso, houve dificuldade em avançar o eletrodo dentro do septo em direção ao RE. A bainha foi, então, avançada para permitir maior sustentação do eletrodo e pode ter, inadvertidamente, produzido um alargamento na entrada e em parte do conduto intraseptal, resultando em menor sustentação e eventual deslocamento do eletrodo. A partir dessa observação (paciente 8), a bainha não foi mais forçada contra o septo interventricular. Não foram observadas outras complicações ou deslocamentos no seguimento de curto prazo (30 dias).

Outro paciente apresentou abscesso de parótida com bacteremia e hemocultura positiva 2 semanas após o implante. O paciente optou por não realizar o explante dos eletrodos e do MP e manter-se em uso contínuo de antibioticoterapia. Após 7 meses de medicação oral, apresentou sinais de infecção da loja quando foi, então, realizada a extração do sistema de MP e eletrodos e implante de MP convencional contralateral após controle adequado da infecção.

## Discussão

Esta é a primeira série brasileira a respeito de estimulação direta do ramo esquerdo. Os resultados sugerem tratar-se de técnica factível e segura para reestabelecer a ativação fisiológica do ventrículo esquerdo em pacientes com indicação de MP.

### Achados relevantes e comparação com resultados da literatura

A estimulação direta do sistema de condução cardíaco através de implante de eletrodo junto ao sistema His-Purkinje em suas porções distais, na área do RE, de forma transeptal a partir do ventrículo direito, demonstrou-se um procedimento seguro, factível e com taxa de sucesso alta, independentemente do local de bloqueio que o paciente apresenta. Nesses pacientes, semelhante ao relatado por outros grupos,^8^ as medidas eletrônicas de sensibilidade e estimulação foram consideradas adequadas com a média da onda R de 12 mV e o limiar de captura do sistema de condução médio de 0,5 ± V × 0,4 ms. Como se trata de uma posição dentro do septo interventricular, não foi observada a inscrição de eletrograma atrial pelo eletrodo 3830 que pudesse interferir na programação de sensibilidade em nenhum dos pacientes. O tempo necessário para o implante do eletrodo e do dispositivo e o tempo de fluoroscopia também estiveram de acordo com os demais achados da literatura e encontram-se muito próximo aos dados dos implantes convencionais.^12^

A estimulação direta do sistema de condução do coração pode ser obtida por meio da estimulação direta do feixe de His ou do RE do sistema His-Purkinje. O feixe de His apresenta importante variação anatômica em sua localização fluoroscópica, curta extensão de sua porção estimulável e localização peritricúspide usualmente no ápice do triangulo de Koch, junto ao anel atrioventricular direito. Por essa razão, não raramente, medidas eletrônicas desfavoráveis à estimulação cardíaca são obtidas. A onda R baixa, um limiar de estimulação mais alto que o da estimulação muscular convencional e a presença de *farfield* do eletrograma atrial no canal designado ao feixe de His podem resultar em depleção precoce da bateria do MP, *oversense* da atividade atrial intrínseca com possibilidade de inibição da estimulação ventricular/hissiana e impor dificuldades para a programação adequada do MP.^13 - 16^ Além disso, a técnica de estimulação do feixe de His é mais demorada que a técnica de implante de MP tradicional, o que pode, eventualmente, aumentar o risco de infecção.^17^ Na estimulação direta do RE, uma estrutura mais larga e ramificada que o feixe de His, por outro lado, a situação se inverte. O RE é maior que o feixe de His, sendo tecnicamente mais fácil e reprodutível obter a estimulação do sistema de condução através dessa abordagem. Teoricamente, a estimulação direta do RE é tão fisiológica para o ventrículo esquerdo quanto a estimulação do feixe de His e apresenta melhores parâmetros eletrônicos de sensibilidade, estimulação e maior durabilidade da bateria do MP devido ao limiar de captura menor que o posicionamento do eletrodo no septo proporciona.^18^

Outra observação do presente estudo sugere que duração do complexo QRS após estimulação do RE varia de acordo com o QRS pré-implante: pacientes com complexo QRS alargado apresentam um estreitamento após o implante, devido à correção dos bloqueios de ramo subjacentes, enquanto pacientes com QRS estreito apresentam um pequeno alargamento, devido ao traçado eletrocardiográfico resultante, semelhante ao distúrbio de condução pelo ramo direito (ver Figura 5 ). Isso provavelmente ocorre porque, apesar da ativação do ventrículo esquerdo dar-se principalmente pelo sistema de condução intrínseco – com pequena contribuição muscular (captura não seletiva) –, a ativação do ventrículo direito acontece após condução retrógrada até o feixe de His seguida de ativação anterógrada pelo ramo direito ou, quando a condução retrógrada não estiver presente, de forma passiva a partir do septo interventricular. Nessa técnica de estimulação, o objetivo principal é manter a ativação fisiológica do ventrículo esquerdo. Por esse motivo, utilizamos como um dos critérios de sucesso o TAVE e não a duração do complexo QRS após implante (tempo total de ativação ventricular esquerda e direita).

**Implicações clínicas:** A estimulação direta do ramo esquerdo pode diminuir ou talvez evitar a dissincronia causada pela estimulação muscular convencional do ventrículo direito e reduzir as taxas de miocardiopatia induzida pelo MP.^19^ Nesses casos, a utilização de um MP de câmara única (como em pacientes com fibrilação atrial permanente) ou câmara dupla (pacientes em ritmo sinusal) poderia manter a sincronia intra e interventricular sem necessidade de um eletrodo adicional para o seio coronário, bem como os custos de um gerador de TRC. Esse tipo de estimulação evitaria ainda os inconvenientes da estimulação através do seio coronário, como tempo maior de procedimento e de fluoroscopia para acesso aos ramos posterolaterais, uso de contraste endovenoso, estimulação frênica e deslocamento do eletrodo do seio coronário em situações de miocardiopatia induzida pelo MP. Os pacientes submetidos à estimulação do feixe de His proximal que apresentam elevado limiar de captura durante o período de seguimento pós-operatório ou dificuldade na programação devido a interferências do eletrograma atrial e onda R baixa e que necessitam da estimulação do sistema His-Purkinje encontram uma ótima alternativa na estimulação direta do RE, conforme alguns dos pacientes deste estudo.

Outro cenário clínico relevante é que o implante distal ao feixe de His com captura direta do RE pode ser utilizado em pacientes após a manipulação cirúrgica ou por cateter da valva aórtica (TAVR), pois esses procedimentos podem estar relacionados a importante dano ao sistema de condução proximal, potencialmente tornando a estimulação do feixe de His desafiadora ou até mesmo impossível. Há evidências de que a disfunção ventricular relacionada à estimulação convencional do ventrículo direito pode ser mais frequente nesses pacientes, justificando a implementação de terapias destinadas à ressincronização ventricular de forma precoce nesses casos.^20^

Da mesma maneira, a estimulação do RE pode ser preferencialmente utilizada em pacientes com fibrilação atrial submetidos à ablação do nó atrioventricular, reduzindo o risco de miocardiopatia induzida pela estimulação ventricular convencional.^11^

**Implantes sem sucesso:** Os dois pacientes nos quais não obtivemos sucesso apresentavam importante aumento das cavidades ventriculares direita e esquerda, o que pode ter dificultado o posicionamento da bainha no ângulo correto junto ao septo interventricular, uma vez que a bainha foi desenhada para facilitar o acesso ao feixe de His em coração de tamanho normal. A curva fixa da bainha pode não proporcionar, nesses casos, o alcance necessário para permitir acesso ao septo interventricular. Nessas situações, na tentativa de conferir mais estabilidade e sustentação ao conjunto bainha-eletrodo, torque excessivo na manipulação pode ter justificado o insucesso nesses casos. Em pacientes previamente submetidos a implantes intravasculares, a redução do calibre do trajeto venoso por fibrose e/ou aderência em virtude da presença de eletrodos antigos pode levar à importante redução da mobilidade da bainha, essencial para o adequado posicionamento do conjunto e fixação do eletrodo.

### Limitações

O procedimento de estimulação do RE realizado neste trabalho utiliza um eletrodo que não foi desenhado especificamente para estimulação transeptal, porém obtivemos uma taxa de sucesso de 96,2%. Trata-se de um trabalho retrospectivo, avaliando um pequeno número de pacientes em um único centro médico, o que não permite a comparação do efeito clínico dessa abordagem com o implante de MP convencional ou do ressincronizador cardíaco. Apesar de ser uma técnica promissora e de provável grande utilidade clínica, faz-se necessária uma avaliação mais detalhada a longo prazo antes que tal abordagem possa ser incorporada à rotina de implantes em substituição ao MP convencional ou aos ressincronizadores.

## Conclusão

A estimulação do sistema de condução cardíaco por meio da estimulação do ramo esquerdo do sistema His-Purkinje é uma técnica segura e exequível, com alta taxa de sucesso, realizada com tempo de procedimento e fluoroscopia baixos, e que resulta em tempo de ativação ventricular esquerdo curto e medidas eletrônicas adequadas.
