# Supplementary material for: Estimulação do Ramo Esquerdo do Sistema His-Purkinje: Experiência Inicial
Source: Arq Bras Cardiol. 2021 Nov 17;118(2):505–16. [Article in Portuguese] doi: 10.36660/abc.20201085 (PMC8856673; doi:10.36660/abc.20201085)
Supplement: Translation

# Translation: Left Bundle Branch Pacing of His-Purkinje Conduction System: Initial Experience

**Authors:** Alexander Romeno Janner Dal Forno, Caique M. P. Ternes, João Vítor Ternes Rech, Helcio Garcia Nascimento, Andrei Lewandowski, Grazyelle Damasceno, Andre d’Avila

**Affiliations:** 1 Brazil Hospital SOS Cardio, Florianópolis, SC – Brazil

**Keywords:** Artificial Pacemaker, Artificial Cardiac Pacing, Electric Stimulation Therapy

## Abstract

**Background:**

Conventional right ventricular pacing increases the risk of atrial fibrillation and heart failure in pacemaker patients. Stimulation of the left bundle branch (LBB) of the His-Purkinje system can prevent the unwanted outcomes of right ventricular pacing.

**Objective:**

To retrospectively analyze the intraoperative outcomes, electrocardiographic and clinical data from the initial follow-up of patients submitted to stimulation of the LBB.

**Methods:**

The electronic parameters of the implant and of possible early complications of 52 consecutive patients submitted to stimulation of the conduction system were evaluated. The adopted significance level was 0.05.

**Results:**

Fifty-two patients underwent left bundle branch stimulation, with 50 successful procedures; 69.2% of the patients were male, and the median and interquartile range of age at the time of implantation was 73.5 (65.0-80.0) years. The pre-implant QRS duration was 146 (104-175) ms and 120 (112-130) ms after the procedure. The left ventricle activation time was 78 (70-84) ms. The R-wave amplitude was 12.00 (7.95-15.30) mV, with a stimulation threshold of 0.5 (0.4-0.7) V x 0.4 ms and impedance of 676 (534-780) ohms. The procedure duration was 116 (90-130) min, and the fluoroscopy time was 14.2 (10.0-21.6) min.

**Conclusion:**

Cardiac stimulation of the His-Purkinje conduction system through the stimulation of the left bundle branch is a safe and feasible technique. In this study, it showed a high success rate, with low procedure and fluoroscopy periods, achieving adequate electronic measurements.

## Introduction

Right ventricular stimulation is the most used method of stimulation around the world for the correction of atrioventricular (AV) conduction disorders. However, this type of stimulation increases the risk of atrial fibrillation, may worsen the heart failure functional class (HFFC) and increase the need for hospitalization due to heart failure (HF) in up to 20% of the patients in 4 years.^1 - 3^ The risk of these adverse events increases when ventricular stimulation is necessary > 40% of the time, and among patients with ventricular disfunction previous to the implant, especially when the duration of the stimulated QRS exceeds 150 ms. Several alternative sites have already been explored in the attempt to prevent the harmful effects of muscular ventricular stimulation, without real proof of its clinical or ecochardiographic benefits.^4^ In these patients, cardiac resynchronization therapy (CRT) may improve or reverse the harmful effects of right ventricular stimulation by reducing the intra and interventricular disynchronization, as it occurs among patients with left bundle branch block (LBBB).^5^

As an alternative to conventional CRT, the His-Purkinje conduction system can be used in any of its portions, both initial and in the His or the more distal axis, such as the left bundle branch (LBB). In the stimulation of the His axis, the right ventricular electrode is fixated close to the apex of the Koch’s triangle, allowing the selective capture – or not – of the proximal His-Purkinje system. Therefore, by recruiting the heart conduction system, it reestablishes the normal physiology of ventricular activation, thus preventing the unwanted effects of conventional stimulation.^3 , 6^ However, this technique is limited. In many cases, the necessary energy to capture the His axis is very high when compared to the necessary energy for the conventional stimulation of the right ventricle, thus resulting in the fast discharge of the pacemaker (PM) battery, especially in patients with intra or infra-hisian blocks and distal LBB block. Besides, the intrinsic ventricular activity measured by the device (R-wave) can have very low amplitude in the His axis position, thus making it difficult to program the PM.^7^

As an alternative to the technique of the Hix axis stimulation, in 2017, Huang et al. described the first case of conduction system stimulation directly in the LBB, in a patient who was ineligible for the physiological stimulation of the His axis.^8^ In this approach, the right ventricular electrode (usually the same electrode addressed to the implant in the His axis) is deeply fixated in the interventricular septum, thus reaching the subendocardial region of the left ventricle, allowing the direct stimulation of the His-Purkinje conduction system by activating the LBB. This stimulation modality results in complex QRS, whose duration is equivalent to that of the His axis stimulation, and lower capture threshold and adequate R-wave, thus facilitating PM programming and allowing the durability of the battery to be similar to that of conventional PMs.^7^

This study aims at presenting the surgical and electrocardiographic immediate result of the early clinical follow-up of the first 52 patients who underwent PM implant to stimulate the conduction system, with the direct stimulation of the left bundle branch of the His-Purkinje system, in an electrophysiology reference center.

## Methods

This is a descriptive, retrospective study in a center to assess the feasibility of an alternative site of stimulation for the His-Purkinje conduction system. We considered 4 inclusion categories for the study: primary implant, secondary implant, high hisian PM threshold (>2.0 V x 1.0 ms) and resynchronization ( Figure 1 ). Primary implants were considered for all patients for whom the initial intention of the intervention was the LBB stimulation of the His-Purkinje system; secondary implants were considered as all of the interventions in which the initial intention was the His axis stimulation, without success due to the high transoperative threshold (threshold > 2.0 V or patients with LBB and threshold > 2.5 V, with block correction).

**Figure 1:** – Flowchart of the distal His-Purkinje system stimulation – left bundle branch. Patients with indication for PM who presented with narrow QRS or node level block, and presenting with His capture thresholds higher than 2V X 1.0 ms, or higher than 2.5V X 1.0ms for the correction of pre-existing branch blocks, were referred for more distal implants with the LBB (secondary implant). Patients with intra or infrahisian block documented by an electrophysiological study were directly sent for distal stimulation (called primary implant). In cases of unsuccess, the patient was referred to conventional septal stimulation or biventricular stimulation. PM: pacemaker; AVB: atrioventricular block; LBBB: left bundle branch block; RV: right ventricle.

Fifty-two consecutive patients were included and underwent a pacemaker implant with LBB stimulation at a reference center. All patients signed the informed consent form before the procedure. This is a retrospective study, with information collected from the medical implant records at Hospital SOS Cardio, performed in accordance with the Declaration of Helsinki and approved by the Ethics Committee of Instituto de Cardiologia do Estado de Santa Catarina, through the Certificate of Appreciation n. 36517420.7.0000.0113, with favorable opinion, n. 4.293.667. The following were assessed: acute success in the procedure, early complications and the need for reintervention. The patients included in this study had indication for PC implant, according to the Guidelines for Implantable Electronic Cardiac Devices and ACA/AHA/HRS, 2018, and underwent the LBB stimulation procedure between August, 2019, and November, 2020, at a reference medical center.

### Septal electrode placement for left bundle branch stimulation

Access pathway: All of the procedures were carried out under general anesthesia. Fluoroscopy-guided axillary vein puncture, with or without contrast injection in the left cubital vein, was the chosen access pathway. In one of the patients, who has had a PM for 8 years, we observed left subclavian vein total occlusion. In this case, the implant was placed through the right axillary vein, and the electrode was tunneled until the left subpectoral space. In the other patients, the left axillary access carried on without complications.

**Sheath and Electrode for Left Bundle Branch stimulation:** After the venous puncture, a fixed curve Medtronic C315HIS^®^69 cm sheath (Medtronic, Minneapolis – MN) was inserted through the right atrium, assisted by a long guidewire (180 cm). A Medtronic *Select Secure* ^®^electrode, model 3830, was placed, through the sheath, at the level of the right atrioventricular ring, after a discreet counterclockwise rotation. The distal pole of the 3830 *Select Secure* ^®^Medtronic electrode was exposed to allow the unipolar mapping of the His axis electrogram ( Figure 2 ). Exceptionally, electrophysiology catheters were concomitantly used in cases that required an electrophysiological analysis to confirm the block level (2 cases). This position was fixed with fluoroscopy at the right anterior oblique position, 30° (RAO), and maintained as reference while mapping the right septal region. Starting at this position, and after a mild clockwise rotation, the sheath and the 3830 electrode were, then, taken through the tricuspid valve about 1 to 1.5 cm in the inferoapical region, in relation to the position where the electrogram of the His axis had been registered.

**Figure 2:** – Technique to implant the Electrode for the capture of the left bundle branch after an attempt of the His Electrogram Capture – The left superior panel shows infrahisian AVB 2:1 during continuous atrial stimulation, with a 700 millisecond cycle. In conducted beats, it is possible to observe an AH interval = 85 ms, and HV = 77 ms. In the middle and left panel, we observe a decapolar catheter in the coronary sinus (CS), which was used for atrial stimulation, and a quadripolar catheter in the His position, at the right anterior oblique position 30º. This case was conducted together with the performance of an electrophysiological study, and we were able to visualize a decapolar catheter in the coronary sinus. The quadripolar catheter was used as a reference for the placement of the PM electrode in the His axis position – it is possible to observe the current of the lesion in the intracavity electrogram. During the continuous stimulation of the His electrode, selective hisian capture is observed without the correction of the right bundle branch block, with 5 V of energy and wrist width of 1 ms. When we increase the stimulation energy from 5 to 6V, the non-selective capture of His occurs, with the correction of the right bundle branch block and cycle of stimulation of 1000 ms. But the block occurs again when the stimulation cycle is reduced to 600 ms. Therefore (high threshold and persistence of the branch block with heart rate of 100 bpm [600ms]), the choice was for the implant of the same electrode in the septal lowest region (inferior right panel showing a projection in the left anterior oblique position at 30º). It is possible to observe the His electrogram record in the quadripolar catheter and the LBB electrogram in the PM electrode [E1] 44 milliseconds after, suggesting that the block is between the His and the location where the potential LBB was registered. A: atrial electrogram; p: proximal; d: distal.

In the first 10 cases, there was unipolar stimulation in areas that were supposedly favorable to the entry of the septal electrode, defined by the presence of the QRS complex, of the “W” morphology in V1 derivation (and/or narrowing of the QRS with R-wave pattern being prevalent in derivation DI, Rs in derivation DII and rS in derivation DIII – Figure 3 ).^8^ However, according to the authors’ experience, such a morphology of the QRS complex could be obtained in several positions of the interventricular septum in a single patient, whereas in others such a pattern could not be observed in the QRS, even after extensive mapping of the right interventricular septum. Therefore, in the 42 subsequent cases, the selected location for the electrode entry was defined only by the placement of the electrode in the interventricular septum in relation to the reference of the site where a His electrogram had been registered.

**Figure 3:** – Variation of impedance during the placement of the stimulation electrode for the capture of the left bundle branch. It is possible to observe a progressive increase of impedance, from 560 to 760 ohms, followed by a progressive increase in the R-wave in V1. The initial measurement shows the QRS complex in V1, with “w” morphology. Whereas the electrode advances through the interventricular septum towards the subendocardial region of the left interventricular septum, the R’ wave in V1 and the impedance during unipolar stimulation increase progressively. The 12-lead electrocardiogram illustrates the final morphology of the QRS complex.

In these locations, more counterclockwise rotation was applied to the sheath until an angle that was close to 90º between the interventricular septum and the C315HIS^®^sheath could be observed in the left anterior oblique projection 30º (LAO). Once this position was reached, the 3830 electrode was gently clockwise rotated until its fixation and mild penetration of the distal electrode (active fixation mechanism) in the interventricular septum. While progressive insertions of the electrode were visualized, the unipolar stimulation was carried out to assess the duration and morphology of the stimulated QRS complex, the local electrogram (when an intrinsic QRS was available) and the impedance measurement. While the electrode penetrates the interventricular septum, the impedance increases gradually during unipolar stimulation ( Figure 3 ).

The electrode, then, was pushed through clockwise rotation until reaching a depth of about 1 cm, in relation to the distal extremity of the sheath, overlapped to the interventricular septum, in the left anterior oblique position at 30º (LAO). After this point, the 3830 electrode was connected to the electrophysiology polygraph (EP Tracer^®^Schwarzer Cardiotek, NL) and to the pacemaker analyzer for the record of the local electrogram and the R-wave measurement. Morphology, duration of the QRS complex, and impedance variation – which gradually increases while the electrode penetrates the interventricular septum – were compared to those obtained at the entry point of the electrode during stimulation, with 3.0 V x 0.4 ms energy. If there was no criterion to capture the LBB, the electrode would be slowly introduced, with an additional clockwise rotation of one and a half lap, and the measurements would be repeated until these criteria could be obtained.

**Criteria to define Left Bundle Branch capture:** One of the most accepted capture criterion of the left bundle branch, nowadays, is the measurement of time for the complete depolarization of the left ventricle, called left ventricular activation time (LVAT). In terms of electrocardiography, LVAT corresponds to the millisecond interval between the stimulation spike and the R-wave peak in V4, V5 or V6; when lower than 90 ms, it characterizes the fast depolarization of the LV, through a conduction system. In the points where there was complex QRS with morphology suggestive of LBB capture (morphology qR or rsR’ in V1), stimulation was performed with low and high energy (respectively, 1.5 V x 0.4 ms and 10 V x 0.4 ms), in order to observe the additional reduction of the QRS complex duration and LVAT in milliseconds (MS). If the site was considered as inadequate, the electrode would be inserted for a few more millimeters, until there was no reduction of these intervals during high energy stimulation.

**Maneuvers to assess Left Bundle Branch Capture:** LBB capture was defined as the presence of a characteristic electrocardiographic pattern in V1 (qR or rqR’) and at least one of the following criteria: 1) Identification of the LBB potential/Purkinje, preceding the QRS complex, with local potential interval until the surface electrocardiogram between 10-50 ms ( Figure 4 ); 2) LVAT maintenance < 90ms during the unipolar stimulation with high and low energy; 3) progressive change between the non-selective and selective pattern of LBB capture during unipolar stimulation with different levels of energy; 4) change from the non-selective pattern to a muscular septal pattern when too close to the final capture threshold; and finally, 5) extrastimuli testing and pure muscular response or LBB selective capture.^9^

**Figure 4:** – Pacemaker implant for the Capture of the Left Bundle Branch in a patient with TAVB. Female patient with TAVB and escape, with narrow QRS complex (panel A), submitted to PM implant for the capture of the LBB. Panel B shows electrocardiographic leads and the intracavity record of the LBB potential with HV = 22 ms. The position of the PM electrode, placed deeply into the interventricular septum, can be observed in panel C in the left anterior oblique position at 30º. In this location, the R wave was 12 mV, with stimulation threshold of 0.4VX0.4ms, resulting in a narrow stimulated QRS, identical to that of the escape rhythm, as shown by the precordial leads (V1 to V6) in panel D.

**Extrastimuli testing:** The proximity between the threshold stimulation value of the LBB and the heart muscle that occasionally surrounds it may make it difficult to distinguish the LBB capture and the left ventricular septal capture, since the stimulated QRS complex may represent a fusion of the stimulation patterns of both structures. Therefore, we usually use the extrastimuli test described by Jastrzbski.^10^ The test consists of applying, by using an electrophysiology polygraph, a fixed cycle of unipolar ventricular stimulation of eight paced beats in the electrode addressed to LBB, followed by an extrastimulus with progressive shorter coupling. The second form of the test consists on the deflagration of an isolated extrastimulus, also with progressively shorter coupling. The conclusion is that there is LBB capture when there is a change in the morphology of the QRS complex, from a non-selective capture of the LBB to a pure muscular capture or selective capture of the conduction system.^9^ Both are diagnostic answers, as illustrated in Figure 5 . Another way to confirm LBB capture is a threshold test with small wrist width, such as 0.1 or 0.05ms.

**Figure 5:** – Extrastimuli technique through the pacemaker electrode to show the left bundle branch capture. Panel A shows the presence of sinus rhythm with LBBB, and duration of the QRS complex of 136 ms (two initial beats). During the LBB stimulation, it is possible to observe a reduction in the duration of the QRS complex, to 14 ms, with left ventricular activation time (LVAT) of 78 ms. The electrode position can be shown in left anterior oblique projection at 30º, during the contrast injection through the sheath, and a bidimensional transthoracic echocardiogram illustrates the position of this same electrode through the interventricular septum in panel C. Panels D and E illustrate the difference in the morphology of the QRS complex in V1 during the continuous stimulation, followed by extrastimuli with progressively shorter coupling interval. As opposed to the demonstrated in panel E, where a qR complex can be clearly identified, the QRS complex in V1 does not present the characteristic morphology of LBB capture. In panel D, the refractory period of the muscular portion of the interventricular septum had not been reached yet. With the reduction of the extrastimulus coupling in 10 ms, we observe the loss of septal muscular capture and the typical LBB capture pattern. Abbreviations: RV: right ventricle; LV: left ventricle.

**Removal of the sheath and connection of the septal electrode to a generator:** Once the LBB capture is confirmed, a small amount of contrast is injected using a C315 sheath to contrast with the right interventricular septum edge and to register the depth reached by the electrode in the intraseptal route. At this moment, the sheath was retreated until the right atrium, the electronic measurements were repeated and the sheath was finally removed, according to the manufacturer’s orientations.

**Connecting the electrodes to the generator:** In cases of complete atrioventricular block with wide QRS escape, due to the risk of presenting with asystole during intraseptal manipulation, the electrode that was initially destined to the atrium was fixated to the right ventricle and used for temporary ventricular stimulation, and after the implant of the intraseptal electrode, taken to the right auricular appendage. In cases of dual chamber and resynchronization PM, the 3830 electrode was connected to the right ventricular port of the device. In the one case in which the left bundle branch PM was used in a defibrillator, the 3830 electrode was connected to the right ventricular port through a IS1/DF1 connection.

In cases of dual chamber PM or CRT, the atrial and left ventricular electrode (coronary sinus) were placed with the usual technique.

**Statistical analysis:** The variables included in the analysis were: sex, age, comorbidities, underlying cardiopathy, type of bradyarrhythmia, mean follow-up time, duration of pre and post-implant QRS complex, LVAT, type of indication for the procedure, time of procedure and fluoroscopy, as well as the capture threshold (value of the unipolar stimulation threshold x wrist width in Volts), electrode impedance, and R-wave amplitude. The electric parameters of the two patients in which the LBB capture was not possible were excluded from this analysis.

Data normality was assessed using the Shapiro-Wilk test. The continuous variables of the implant parameters presented non-normal distribution and were compared using the non-parametric Wilcoxon test. The results were described as median and interquartile range (IQR) Q1 – Q3 (percentile 25 – percentile 75). A two-tailed p-value < 0.05 was considered as statistically significant. All of the analyses were performed using the R software, version 3.6.2.

## Results

### Patients’ characteristics:

Fifty-two procedures were carried out. The median and IQR of the patients’ age was 73.5 (65.0-80.0) years, and 69.2% were male. Forty patients presented with non-ischemic cardiomyopathy (76.92%), 4 presented with ischemic cardiomyopathy (7.69%), and 8 presented with previous PM-induced cardiomyopathy (15.38%). The characteristic of the type of bradyarrhythmia of the patients is described in Table 1 , and one patient can present with more than one type of bradyarrhythmia. Seven patients with atrial fibrillation (AF) needed a PM to control frequency after complete atrioventricular block (CAVB). In 63.5% of the implants (n=33), the initial intention of the procedure was the primary PM implant to stimulate the LBB. In 13.5% of the patients (n=7), there was an initial attempt to stimulate the His axis, and due to unsatisfactory measures obtained in the transoperative period (stimulation threshold > 2 V x 1 ms and/or R-wave < 2 mV), the choice was for the LBB stimulation – considered as secondary implants. In 7.7% of the patients (n=4), with previous hisian PM, there was an increase in the command threshold > 4,0 V x 1 ms during clinical follow-up; 15.4% of the patients (n=8) did not respond to conventional CRT, and were submitted to LBB stimulation. Success was obtained in 50/52 patients (96.2%), which is similar to the rates shown in the literature.^11^ The data of two patients in which it was not possible to capture the LBB were not included in the following analyses. Seventeen patients had structural cardiomyopathy and left ventricular ejection fraction (LVEF) lower than 50%. Of the implanted devices, 8 were resynchronization (15.4%), 3 were single-chamber PM (5.8%), 3 were dual chamber cardioverter defibrillator (5.8%), and 38 were dual chamber PMs (73.1% of the devices).

**Table 1:** – Demographic characteristics of the patients who underwent left bundle branch stimulation

	Total (N=52)
**Sex, n (%)**	
Male	36 (69.2%)
Female	16 (30.8%)
Age (years), *median (IQR)*	73.5 (65.0-80.0)
SAH, n (%)	39 (75.0%)
DM, n (%)	17 (32.7%)
**HFFC, n (%)**	
NYHA I-II	11 (21.2%)
NYHA III-IV	41 (78.8%)
**Underlying cardiopathy, n (%)**	
Non-ischemic	40 (76.2%)
Ischemic	4 (7.69%)
INduced by previous PM	8 (15.38%)
**Type bradyarrhytmia, n (%)** ^¶^	
Sinus node dysfunction	3 (5.8%)
1st degree AVB	4 (7.7%)
2nd degree AVB	7 (13.5%)
3rd degree AVB	24 (46.2%)
AF + TAVB	5 (9.6%)
Basal LVEF, *median (IQR)*	56.2 (44.8-67.1)
**Indication, n (%)**	
Primary implant	33 (63.5%)
Secondary implant	7 (13.5%)
High threshold – hisian PM	4 (7.7%)
CRT	8 (15.4%)
**Outcime, n (%)**	
Success	50 (96.2%)
Failure	2 (3.8%)

*¶: The patients may present more than one type of bradyarrhythmia. SAH: Systemic Arterial Hypertension; DM: Diabetes Mellitus; AVB: atrioventricular block; AF: atrial fibrillation; TAVB: total atrioventricular block; PM: pacemaker; CRT: Cardiac resynchronization therapy; HFFC: Heart Failure Functional Class; NYHA: New York Heart Association; LVEF: left ventricular ejection fraction.*

**Electronic measurements:** The median and IQR of the duration of preoperative QRS was 146 (104-175 ms). The duration of the QRX complex during LBB stimulation was 120 (112-130) ms ( Figure 6 ), with LVAT of 78 (70-84) ms. The mean duration of the procedure was 116 (90-130) minutes, with mean fluoroscopy time of 14.2 (10.0-21.6) min. The stimulation threshold for LBB capture was 0.5 (0.4-0.7) V x 0.4 ms, with impedance during stimulation of 676 (534-780) Ohms, and R-wave of 12.00 (7.95-15.30) mV. In five patients with CAVB without escape, it was not possible to assess the amplitude of the R-wave.

**Figure 6:** – Duration of the QRS complex before and after the direct stimulation of the left bundle branch. The Wilcoxon non-parametric test was used (p=0.001).

**Unsuccessful implants:** In two (2/52) patients (3.8%) with dilated cardiomyopathy, it was not possible to obtain the LBB capture criteria. In the first case, the patient presented with increased cavity diameter and LVEF of 38%. It was difficult to handle the C315 sheath, thus preventing the mapping of the His axis. After several attempts, fracture of two sheaths and prolonged duration of the procedure and fluoroscopy, we chose to use the conventional resynchronization implant with a left ventricular electrode in the posterolateral branch of the coronary sinus.

In the second patient, with diastolic left ventricular diameter of 59 mm and LVEF of 35%, without previous PM implant and wide QRS complex (infra-hisian CAVB), it was not possible to demonstrate any of the LBB capture criteria despite the difficult placement and apparent adequate position of the ventricular electrode. The electrode was replaced with a conventional active-fixation electrode, placed in the mid-septal region of the right ventricle, due to the possibility of displacement due to the large number of electrode rotations in the attempt to reach the LBB.

**Clinical follow-up:** During follow-up [median and (IQR)] 8.00 (3.25-10.0) months, it was possible to observe the displacement of the septal electrode to the sub-tricuspid portion of the right ventricle in a patient who presented completely normal parameters at the end of the procedure. This patient remained asymptomatic for 30 days when, at the follow-up appointment in the outpatient clinic, a wide QRS complex was observed, and a thoracic X-ray confirmed the displacement. The patient once again underwent the LBB stimulation implant and remained clinically stable, with stable LBB capture parameters until the last follow-up appointment, six months after the reimplant. In this case, there were difficulties to introduce the electrode inside the septum towards the LBB. The sheath was, then, introduced to provide more support to the electrode, and may inadvertently have produced an enlargement in the entrance and in part of the intraseptal conduct, resulting in less support and possible electrode displacement. Considering this observation (patient 8), the sheath was not forced against the interventricular septum. No other complications or displacements were observed in the short-term follow up (30 days).

Another patient presented with parotid abscess, bacteremia and positive blood culture two weeks after the implant. The patient chose not to remove the electrodes and the PM, and to remain with the continuous use of antibiotics. After 7 months of oral medication, the patient presented with signs of infection, and then the pacemaker and the electrodes were removed, followed by the conventional contralateral PM implant after the adequate control of the infection.

## Discussion

This is the first Brazilian series about the direct stimulation of the left bundle branch. The results suggest it is a viable and safe technique to reestablish the physiological activation of the left ventricle in patients with indication for PM.

**Table 2:** – Results of the procedures and electronic parameters of the pacemakers

	Total (N=50)
Preoperative QRS (ms), median (IQR)	146 (104-175)
Postoperative QRS (ms), median (IQR)	120 (112-130)
LVAT (ms), median (IQR)	78 (70 - 84)
Time of fluoroscopy (min), median (IQR)	14.2 (10.0-21.6)
Time for the procedure (min), median (IQR)	116 (90-130)
Threshold (V x 0.4 ms), median (IQR)	0.5 (0.4-0.7)
Impedance (ohms), median (IQR)	676 (534-780)
R-wave (mV), median (IQR)	12.00 (7.95-15.30)

*LVAT: left ventricular activation time.*

**Relevant findings and comparison with results in the literature:** The direct stimulation of the cardiac conduction system through the implantation of an electrode with the His-Purkinje system in its distal proportions, in the LBB region, in a transseptal manner starting in the right ventricle, has proven to be a safe, viable procedure, with high success rates, regardless of the block site presented by the patient. In these patients, similarly to the reports of other groups,^8^ the electronic sensitivity and stimulation measurements were considered as adequate, with mean R-wave of 12mV and mean conduction system capture threshold of 0.5 ± V x 0.4 ms. Since this is a position inside the interventricular septum, it was not possible to observe the inscription of the atrial electrogram by the 3830 electrode that could interfere in the sensitivity programming in any of the patients. The necessary period of time for the electrode and device implant and the time of fluoroscopy were also in accordance with the other findings in the literature, and are very close to the data regarding conventional implants.^12^

The direct stimulation of the heart conduction system can be obtained through the direct stimulation of the His axis or the LBB of the His-Purkinje system. The His axis presents major anatomical variation in its fluoroscopy location, short extension of its stimulable portion, and peritricuspid location usually at the apex of the Koch’s triangle, together with the right atrioventricular ring. Therefore, electronic measurements that are unfavorable to cardiac stimulation are not rarely obtained. The low R-wave, a higher stimulation threshold than that of conventional muscular stimulation, and the presence of “fairfield” of the atrial electrogram in the channel addressed to the His axis may result in the early discharge of the PM battery, the “oversensing” of the intrinsic atrial activity, with possibility of inhibiting the ventricular/hisian stimulation, and the imposition of difficulties for the adequate programming of the pacemaker.^13 - 16^ Besides,the His axis stimulation technique takes longer than the traditional PM implant, which may increase the risk of infection.^17^ However, in the direct stimulation of the LBB, a larger and more branched structure than the His axis, the situation is the opposite. The LBB is larger than the His axis, thus being technically easier and more reproducible to obtain the conduction system stimulation through this approach. In theory, the direct stimulation of the LBB is as physiological for the left ventricle as is the stimulation of the His axis, and presents better electronic sensitivity parameters, stimulation and longer durability of the PM battery due to the lower capture threshold than that provided by the placement of the electrode in the septum.^18^

Another observation in this study suggests that the duration of the QRS complex after the LBB stimulation ranges according to the pre-implant QRS: patients with wide QRS complex present narrowing after the implant, due to the correction of the blocks in subjacent branches, whereas patients with narrow QRS present with minor enlargement due to the resulting electrocardiographic design, similar to the conduction disorder by the right bundle branch ( Figure 5 ). This probably happens because even though the activation of the left ventricle is mainly owed to the intrinsic conduction system – with minor muscular contribution (non-selective capture) – the activation of the right ventricle occurs after a retrograde conduction until the His axis, followed by the anterograde activation through the right bundle branch, or, when there is no retrograde conduction, passively starting in the interventricular septum. In this stimulation technique, the main goal is to obtain the physiological activation of the left ventricle. Therefore, one of our success criteria was the LVAT, and not the duration of the QRS complex (total time of left and right ventricular activation).

**Clinical Implications:** The direct stimulation of the left bundle branch may reduce, or maybe prevent the dyssynchrony caused by the conventional muscular stimulation of the right ventricle, as well as reduce the rates of cardiomyopathy induced by the PM.^19^ In these cases, the use of a single chamber (such as in patients with permanent atrial fibrillation) or dual chamber PM (patients in sinus rhythm) could maintain the intra and interventricular synchrony without the need for an additional electrode for the coronary sinus, as well as the costs of a CRT generator. This type of stimulation would prevent the inconveniece of the stimulation through the coronary sinus, with longer period for the procedure and fluoroscopy to access the posterolateral branches, use of intravenous contrast, phrenic stimulation and displacement of the electrode in the coronary sinus in situations of pacemaker induced cardiomyopathy. Among the patients submitted to the proximal His axis stimulation who present with high capture threshold during the postoperative follow-up period, or difficulties in the programming due to the interferences of the atrial electrogram and low R-wave, which require the stimulation of the His-Purkinje system, the direct stimulation of the LBB is a great alternative, according to some patients in this study.

Another relevant clinical scenario is that the distal implant to the His axis, with direct capture of the LBB, can be used in patients after surgical manipulation or through a transcatheter aortic valve replacement (TAVR), because these procedures can be related to major damage to the proximal conduction system, potentially making the His axis stimulation more challenging, or even impossible. There is evidence showing that the ventricular dysfunction related to the conventional stimulation of the right ventricle can be more frequent in these patients, thus justifying the implementation of therapies addressed to the early ventricular resynchronization in these cases.^20^

Likewise, the stimulation of the LBB can be preferably used in patients with atrial fibrillation submitted to the AV node ablation, thus reducing the risk of cardiomyopathy induced by conventional ventricular stimulation.^11^

**Unsuccessful implants:** The two patients that were not successful presented with major increase of the right and left ventricular cavities, which may have made it difficult to place the sheath in the correct angle near the interventricular septum, once the sheath was designed to facilitate the access to the His axis in a normal-sized heart. The fixed curve of the sheath may not provide, in these cases, the necessary reach to allow access to the interventricular septum. In these situations, in the attempt to provide more stability and support to the sheath-electrode set, excessive torque in the manipulation may have justified the lack of success in these cases. In patients who had previously undergone intravascular implants, the reduction of the venous route caliber due to fibrosis and/or adherence due to the presence of old electrodes, may lead to a major reduction in the sheath mobility, which is essential for the adequate placement of the set and fixation of the electrode.

### Limitations

The LBB stimulation procedure carried out in this study uses an electrode that was not specifically designed for transseptal stimulation; however, we obtained a 96.2% success rate. This is a retrospective study that assesses a small number of patients in a single medical center, which does not allow the comparison of the clinical effect of this approach with the conventional PM implant or the cardiac resynchronization. Despite being a promising technique, with probable great clinical use, it is necessary to assess it in more detail and in the long term before this approach can be incorporated to the routine of implants to replace conventional PM or resynchronization.

## Conclusion

The stimulation of the cardiac conduction system through stimulating the left bundle branch of the His-Purkinje system is a safe and viable technique, with high success rates, carried out with low time of procedure and fluoroscopy, and results in short time of left ventricular activation and adequate electronic measurements.
